# The Bibliometric Analysis of the Sustainable Influence of Physical Education for University Students

**DOI:** 10.3389/fpsyg.2021.592276

**Published:** 2021-03-04

**Authors:** Dekai Xu, Yingying Zheng, Yunli Jia

**Affiliations:** ^1^General Graduate School, Hoseo University, Asan, South Korea; ^2^College of Physical Education and Health, Wenzhou University, Wenzhou, China; ^3^Graduate Institute of Physical Education, National Taiwan Sport University, Taoyuan, Taiwan; ^4^Dance Academy, Northwest Minzu University, Lanzhou, China

**Keywords:** sustainable influence, physical education, art department, sports participation, mental health promotion

## Abstract

With the awakening of people's health consciousness, the concept and practice of health promotion has become the main target of health policies throughout the world. In this study, the relationship between physical education and health promotion was examined. Art students from a university in Taoyuan were selected for research, and a total of 320 questionnaires were issued. Invalid and incomplete questionnaires were eliminated, with a total of 227 valid questionnaires. Finally, the LISREL (Linear Structural Relations) model was used to analyze the correlation between various factors and health promotion. The results of the model analysis show that in terms of basic fit, the three factors of physical education (course design, teaching content, and activity design) have a high correlation with the influence of physical education, reaching a significant level (t > 1.96, *p* < 0.05). In terms of influence on sports participation, the three factors (physiological factors, psychological factors, social factors) of sports participation reached a significant level (t > 1.96, *p* < 0.05). The two factors of health promotion (physiological health, and mental health and practical ideas) have a high correlation with the influence of health promotion, reaching a significant level (t > 1.96, *p* < 0.05). In terms of overall mode fit, the overall mode fit standard χ^2^/Df was 1.344, less than the standard value of 3 or less, and the RMR value was 0.007, indicating that the χ^2^/DF and RMR result standards were appropriate, and the chi-square value was very sensitive to the sample size. Therefore, there was a positive correlation among physical education, sports participation, and health promotion. In conclusion, physical activities can improve the human body's immune function, reduce the symptoms of chronic diseases, and positively promote health. The research result is important for emphasizing the benefit of sports to art students, and provides reference for improving the quality of school physical education, and the physical and mental health level of people in Taiwan.

## Introduction

With the change of disease patterns and the awakening of people's health awareness, the concept and practice of “health promotion” has become the main work item of health policies around the world. On the part of sports, many scholars have confirmed that exercise can improve and promote human health. Exercise is a dynamic, positive, and beneficial health behavior. Many studies have confirmed that exercise is helpful to the physical and psychological aspects of an individual.

In school education, nothing is more relevant to the human body than physical education; physical education is the education of physical activity, and the starting point of sports development, and the first arena that physical exercise education can be implemented. However, physical education is often confronted with the lack of sports facilities, restrictions on curriculum and rigid curriculum, and many other deficiencies in physical education, seriously questioning and challenging the quality of school physical education. Fixed time and fixed space give the interaction between teachers and students and students' learning a lack of flexibility. To a certain extent, colleges and universities are deepening the reform of university students' physical education. They need to create a high-quality internal and external environments for students. To enhance the general image of colleges and universities and improve the overall strength of colleges and universities, it is necessary to make greater efforts in the investment of professional teachers and construction funds. Colleges and universities must realize the necessity and importance of deepening the reform of physical education, pay more attention to this mission, and increase the input of teachers and funds. At the same time, colleges and universities should regard physical education as a practical subject, appropriately expand the sports training venues, and ensure that sports training venues are comprehensive and diverse. From the perspective of educational psychology, the cultivation of students' thinking ability can change students' understanding of sports. The purpose is to focus on training students that major in physical education to use the theoretical knowledge they have learned to conduct scientific research, and to improve their social adaptability and humanistic quality. However, at present, there are few studies on the correlation between the sports participation of art students and their health level, especially those studies that obtain specific data results through model analysis to provide evidence for the research on the promotional relationship between sports and health.

Therefore, this study explores the influence of physical education on the sports participation and health promotion of art students. The innovation lies in analyzing the relationship between the sports participation of art students and health promotion through model and factor analysis. It is expected to enhance the emphasis of physical education, the quality of school physical education, and improve the physical and mental health, quality of life, and life satisfaction of Taiwanese people. In addition, this paper will study the relationship between physical education and sports participation and the relationship between physical education and health promotion.

## Literature Review

According to Chen et al. (2016), physical education refers to a purposeful, planned, and organized educational process carried out through physical activity and other complementary means. According to Jang et al. (2017), physical education itself is a complete system and can be divided into two categories: general physical education and specialized physical education. Its basic characteristics are an outstanding educational nature and a pedagogical nature. Physical education takes teaching as the main approach, classroom teaching or specialized counseling as the main form, and physical exercise and health care as the main means. According to Aadland et al. (2017), physical education refers to the goal of cultivating students' leisure knowledge, providing opportunities for student physical activities and improving their motor skills. According to Page et al. (2017), since the formation of the modern education system, sports has been an important means of school education and an important part of the school curriculum. According to Edwards et al. (2017), in the development process of the past two centuries before the middle of the last century, the role of humanities education in early school sports became gradually weakened, and the scientific tendency of the curriculum and the discipline-centered tendency increasingly strengthened, which was embodied by that “enhancing physique” and “skill teaching” had gradually became the main purpose of school sports, and even became the sole purpose in practice; early school physical education for the perfection of the educatee's personality gradually became the perfection of the educatee's body (Didehbani et al., 2016).

According to Huang et al. (2018), physical education should include the following facets:

1. Curriculum design: Through flexible curriculum design and content planning, the spirit of the value orientation of various physical education courses can be combined, coping with the actual needs of teaching, but can also maximize the benefits on the overall development of students' minds and bodies in the enrichment of teaching content and promotion of teaching functions.
2. Teaching content: In the application of teaching materials, there should be multiple choices and some flexibility. Through teachers' reorganization of the curriculum, a complete and flexible teaching unit will be prepared for the textbooks with the same nature and difficulty, which will contribute to the progress of teaching activities.
3. Activity design: The change of activities is the secret to maintaining students' interest in learning and the main condition for fun teaching.

Gao et al. (2017) defined sports participation as “sports activities that are arranged to participate in fun.” Lee et al. (2018) defined sports participation as “an activity that is beneficial to the body and mind and has positive benefits for re-creation at leisure without working.” Sackett and Edwards (2019) pointed out that sports participation was a freely chosen, free-spirited physical activity or movement in free time, ranging from physical exercise to recreational sports. Tomaz et al. (2019) considered that sports participation was a sporty leisure activity combined with sports and leisure; not only could people relax, forget worries, and get rid of their unchanging lifestyle, but it also had entertainment, satisfaction, social, and health improvement functions, so it was a unique activity that other types of leisure activities could not compete with. Bishopa and Pangelinan (2018) defined sports participation as a physical exercise or recreational sport that was freely chosen in free time, and leisure sports could make people happy, fun, and healthy. Wasenius et al. (2018) believed that sports participation could often make people have a good experience in the process of participation, and thus promote balanced life experiences, improve life connotation, and improve the quality of life. Advocating the spirit of the national movement and cultivating the habit of everyone's movement has become an important policy priority for modern countries. Ho et al. (2017) pointed out that sports participation referred to individuals that continued to engage in dynamic sports or activities that were beneficial to physical and mental health through physical activities in free time, and perceived the self-psychological feelings of leisure sports (Herrmann et al., 2017).

According to Chang and Gu (2018), the motivation of sports participation can be roughly divided into three factors:

1. Physiological factors: Good health, good shape, sports skills, and sports knowledge.
2. Psychological factors: The sense of accomplishment, self-challenge, leisure and entertainment, ability performance, relieving stress, and stimulating and gaining.
3. Social factors: Friendship, peer relationship, achievement development, and further education assistance.

## Research Hypotheses

Guo and Goh (2016) pointed out that the most direct and significant value of physical education curriculum for students' growth was to promote sports participation and physical fitness. Menin et al. (2018) believed that such values were mainly expressed in two ways; on the one hand, it was reflected in the changes in body shape and function through participation in sports, so that students can enhance physical fitness and improve their health through participation in sports activities under the guidance of teachers; on the other hand, it is reflected in the changes in sports awareness and behavior through sports participation. Through long-term sports practice and experience, students can not only form correct concepts of physical activity and health, but also enhance the consciousness of self-care and gradually develop the behavioral habits and lifestyles involved in sports. Huang et al. (2017) pointed out that physical education could encourage individuals to choose suitable leisure sports, and have the function of promoting good health, molding their bodies, tempering strong will, establishing self-confidence and comfortable mood, by which life could be adjusted, interpersonal relationships could be improved, and people would be full of vitality. Therefore, this study establishes the following hypothesis:

H1: Physical education has a significant positive correlation with sports participation.

Huang et al. (2017) pointed out that the principle for modern human health promotion was to do more exercises. In addition to physical activities at home, as long as people can participate in leisure activities during leisure time and accumulate enough physical activity every week, they can achieve a highly efficient return on health investment. Participation in recreational activities contributes to body metabolism, adjusts the body and mind, and improves health. Çakiroglu and Gökoglu (2019) believed that in addition to physical fitness, promoting development, providing physical and mental relaxation, improving work efficiency, competition, and rehabilitation, leisure sports participation could have functions in activities such as psychology, learning, social functions, and social system treatment. Naslund et al. (2016) held that engaging in recreational activities could improve the feelings of wellbeing, especially in terms of anxiety, stress reduction, and vitality improvement. Chang and Gu (2018) believed that in addition to having positive effects on physical wellbeing, recreational sports could also improve psychological wellbeing. These positive effects are of course included in diseases such as osteoporosis, hypertension, coronary heart disease, and cancer. Regular physical activity or exercise has a significant effect on the prevention of diseases. Therefore, this study establishes the following hypothesis:

H2: Exercise participation has a significant positive correlation with health promotion.

The process of physical education is a process of constantly facing setbacks and overcoming difficulties. In this process, students can experience setbacks and difficulties repeatedly, thereby improving an anti-frustration ability and emotional adjustment ability, and fostering their brave and tenacious will quality. Ecological sports education enables ordinary citizens to join the movement participants, not only to fully integrate and participate in society, but also to improve health promotion, reduce the burden of government medical care and expenditure of social resources, and make the general people develop the habit of using various types of leisure sports to express psychological pressure, which not only slows down intelligence loss and disability, but also effectively promotes health promotion of the general public. Therefore, this study establishes the following hypothesis:

H3: Physical education has a significant positive correlation with health promotion.

The purpose of establishing the above three hypotheses is to verify whether the sports participation of art students is positively correlated with health promotion.

## Research Method

### Analysis of Physical Education Needs of Art Students Based on Educational Psychology

For art students in colleges and universities, they usually do not pay enough attention to physical education and participate in activities. In fact, students do not hate sports activities or physical education, but feel bored or even disgusted with the contents and teaching methods of some physical education teachers in class. Currently, educational psychology is also reflected in the physical education of higher education. For example, according to the training objectives and the characteristics of students, psychological quality training courses are offered in different stages and levels, including social psychology, sports psychology, and rehabilitation psychology (Wang et al., 2017). These courses try to break the traditional psychological education mode of university students (that is, taking psychological problems as a special topic, discussing a specific issue, and proposing solutions) and put forward eight modules of practical activities, including cognitive development, emotional stability, will optimization, personal perfection, learning adaptation, interpersonal harmony, career adaptation, and mental health education activities for psychological barriers (Zheng and Liu, 2020).

The improvement of sports skills requires a lot of practice. However, this kind of learning theory limits students' learning to conditional reflection and completely ignores students' subjective initiative, logical thinking ability and theoretical knowledge. This is one-sided. Cognitive learning theory is opposite to habit acquisition theory. Cognitive learning theory is a learning theory that explores learning rules by studying human cognitive processes. It holds that human acquire and exchange information through the process of perception, attention, memory, comprehension, and problem-solving. The students with learning difficulties in sports theory refer to the students who are weak in theory learning, difficult to accept, low in psychological quality, and tired of the sports theory course. In this regard, Shen et al. (2018) proposed that a wearable head mounted display (HMD), as a device that can provide users with virtual reality experience, can break through the traditional teaching style and improve teaching experience when applied in physical education. To some extent, the lack of learning motivation of art students may be due to the fact that some of their low-level needs (love and self-esteem) are not fully met. When students with learning difficulties in sports theory try to study and master movement skills, because of the lack of theory, the learning process cannot be supported. They often make great efforts but with little effect, so they will have a sense of loss. At this time, teachers need to enhance communication to promote the comprehensive and individual development of each student majoring in sports. They should pay attention to the art of praise and criticism. They should be tolerant but not indulgent, and strict but not harsh. Shen and Ho (2019) proposed a hybrid bibliometric method combining direct citation network analysis and text analysis in order to improve the teaching effect of colleges and universities. In the teaching of physical education theory, it can summarize the accumulation of knowledge and make it easier for students to accept and understand new knowledge. In addition, physical education is different from other theoretical disciplines. It needs interactive education and the application of new technology in teaching activities can meet the psychological needs of students and improve the quality of teaching. In this regard, Wu et al. (2019) proposed a new class response system (CRS) and mobile device technology, which may help teachers create a student-centered interactive classroom. For university physical education, it can further promote students' divergent thinking and active learning (Ho et al., 2017).

### Research Object

In this study, students of the art department of a university in Taoyuan City were selected as the research objects. The content of the questionnaire includes two aspects: students' participation in sports teaching activities and their cognition of physical education and curriculum. A total of 320 questionnaires were sent out. After removing the invalid and incomplete questionnaires, there were 227 valid questionnaires, with a valid recovery rate of 71%. The information collected from the questionnaire is only used for data analysis, and will not affect personal information security and privacy protection.

### Method Mode

The LISREL model combines factor analysis and path analysis in traditional statistics with econometric equations. It can analyze the relationship between multiple factors and multiple causal paths simultaneously, including the relationship between latent variables and measured variables, and improve research accuracy. The LISREL model extracts factors from interdependent and highly correlated indicators to form an independent factor and incorporate this factor into the analysis process, which makes up for the deficiencies of multiple regression analysis. Path analysis can calculate direct effects and indirect effects between significant variables. If latent variables cannot be calculated, the LISREL model can enhance researchers' in-depth understanding of causality and facilitate causality analysis. Furthermore, systematic professional knowledge is included in the model design process, which breaks through the traditional search for variable relationships that depend too much on mathematical perspectives. The complete LISREL covers a set of variable systems, establishes regression relationships between potential factors through this system, as well as relationships between potential factors and observed variables. Therefore, the complete LISREL model contains a measurement equation and a complete structural equation. The equations are:

(1) $$\begin{array}{r} \eta =\text{B}\eta +\Gamma \xi +\zeta \end{array}$$

(2) $$\begin{array}{r} x=\Lambda_{x}\xi +\delta \end{array}$$

(3) $$\begin{array}{r} y=\Lambda_{y}\eta +\varepsilon \end{array}$$

In Equations (1)–(3), η denotes the internal latent variable, ξ denotes the external latent variable, ζ represents the potential interference, *x* represents the external observation variable, *y* refers to the internal observation variable, Λ_*x*_ refers to the coefficient matrix describing the relationship between *x* and ξ, Λ_*y*_ refers to the coefficient matrix describing the relationship between *y* and η, δ stands for the measurement error of the external observation variable, ε stands for the observation error of internal observational variables, B indicates the regression coefficient matrix of internal and internal latent variables, and Γ m indicates the regression coefficient matrix of external and internal latent variables.

Models based on relevant theories need to be tested further, which can be based on overall model fitting (that is, quality other than the model) and model quality. Commonly used fitting evaluation indicators for detecting overall model fitting include:

1. Chi-square value test, the most basic measurement indicator for overall fitting. Its equation is:
  (4) $$\begin{array}{r} \chi^{2}=\left(N-1\right)\times F \end{array}$$
  In Equation (4), *F* represents the minimum value of the fitting function, and *N* represents the number of experiments. A larger chi-square value indicates a poorer fitting between the model and the data. On the contrary, a smaller chi-square value indicates a better fitting.
2. The Goodness of Fit Index (GFI) can measure the overall fitting of the model to the data. Its equation is:
  (5) $$\begin{array}{r} GFI=1-\frac{F_{\min}}{F_{0}} \end{array}$$
  In Equation (5), *F*_0_ and *F*_min_ respectively refer to the value of the objective function before and after the model is fitted. The Adjusted GFI (AGFI) can also measure the fitting effect, and its equation is:
  (6) $$\begin{array}{r} AGFI=1-\left[\left(k/df\right)\left(1-GFI\right)\right] \end{array}$$
  (7) $$\begin{array}{r} k=\frac{1}{2}\left(p+q\right)\left(p+q+1\right) \end{array}$$
  (8) $$\begin{array}{r} df=\frac{\left(p+q\right)\left(p+q+1\right)}{2-t} \end{array}$$
  In Equations (6)–(8), *p* + *q* denotes the number of measurable variables, *df* stands for the chi-square degree of freedom, and *t* stands for the minimum number of parameters that must be considered for all paths. The values of GFI and AGFI are between 0 and 1. The larger the index value, the better the fitting effect.
3. Root Mean square Residual (RMR) is the square root of the residual mean between the measured matrix and the estimated matrix. The smaller the value, the better the fitting of the model. If the value is less than 0.05, the fitting effect will be better.
4. Incremental Fit Index (IFI) refers to the increment of the chi-square value between the independent model and the hypothetical model. The closer the value is to 1, the better the fitting of the model.

The internal quality evaluation indicators used by LISREL include:

1. The Squared Multiple Correlation (SMC) of a single apparent variable is equivalent to the R^2^ value of the apparent variable and the latent variable, and the value should be >0.5.
2. Cronbach's α coefficient represents the average of reliability coefficients obtained by all possible item division methods, which can describe the component reliability value (ρ) of latent variables and should be >0.6.
3. The calculation mode of average variance extracted from potential variables is to take sum of the apparent variables R^2^ of a potential variable dividing the number of apparent variables, indicating how many percentages of potential variables can be measured by the apparent variables. It is recommended that the valuse should be >0.5.

### Factor Analysis Approach

In this study, the factor analysis approach was made through a qualitative analysis method, also known as the empirical analysis method. Based on previous research experience and the research needs of the target object, the factors that need to be analyzed were selected by researchers. The factor analysis method uses the system indicator system to analyze the response degree of each factor in the change of the phenomenon. It can transform a set of variables that reflect the nature, state, and characteristics of things into simple essential characteristic factors that reflect the internal connections of things. The factor analysis approach was simple and convenient to implement, but required users to have strong research ability and rich research experience. Besides, it can accurately select the most suitable analysis factor, yet this method also has the disadvantage of greater subjective influence. Therefore, the factor analysis approach was adopted in this study to analyze the relationship between health promotion and physical education (Zheng and Ke, 2020).

The main steps of factor analysis are as follows: (1) the data samples are standardized. (2) The correlation matrix R of the samples is calculated. (3) The eigenvalue and eigenvector of the correlation matrix R are calculated. (4) The number of main factors is determined according to the cumulative contribution rate required by the system. (5) The factor load matrix A is calculated. (6) The factor model is determined. (7) According to the above calculation results, the system is analyzed.

The factor analysis model is as follows:

(9) $$\begin{array}{r} X=\mu +LF+e \end{array}$$

In Equation (9), X is the vector of the measured value, μ is the *p* × 1 vector of the mean value, L is the *p* × *m* matrix of the load, F is the *p* × *m* vector of the common factor, and e is the *p* × 1 vector of the residuals. Here, p is the number of measurements of the topic or item, and m is the number of common factors. F and e are assumed to be independent, and each F is independent of each other. The mean value of F and e is 0, and the orthogonal factor model is obtained by assuming the independence of F.

The specific content of factor analysis includes: physical education, sports participation and health promotion, and the relationship among the three.

## Results

### Factor Analysis

The analysis of the factors in this study is shown in Table 1. After the factor analysis of the physical education scale, a total of three factors were extracted. The first factor was the “course design” (characteristic value = 2.183, α = 0.86). The second factor was “teaching content” (characteristic value = 1.662, α = 0.81). The third factor was “activity design” (feature value = 1.345, α = 0.83). The cumulative variation of the three factors accounted for 80.244%; after the factor analysis of the sports participation scale, a total of three factors were extracted. The first factor was the “physiological factor” (characteristic value = 2.533, α = 0.85). The second factor was the “psychological factor” (characteristic value = 2.275, α = 0.84). The third factor was the “social factor” (characteristic value = 1.944, α = 0.87). The cumulative interpretation variable of the three factors accounted for 73.916%; the health promotion scale was extracted by factor analysis, and two factors were extracted. The first factor was “physiological health” (characteristic value = 3.611, α = 0.92). The second factor was “mental health” (characteristic value = 2.737, *a* = 0.91). The common cumulative interpretation of the two factors accounted for 85.447%.

**Table 1:** Factor analysis table.

**Variable**	**Factor**	**Characteristic value**	**α**	**Cumulative interpretation of variable**
Physical education	Course design	2.183	0.86	80.244
	Teaching content	1.662	0.81	
	Activity design	1.345	0.83	
Sports participation	Physiological factors	2.533	0.85	73.916
	Psychological factors	2.275	0.84	
	Social factors	1.944	0.87	
Health promotion	Physiological health	3.611	0.92	85.447
	Mental health	2.737	0.91	

### Related Analysis

It can be seen from Table 2 below that physical education, sports participation, and health promotion are significantly correlated. These results show that H1, H2, and H3 have initial support.

**Table 2:** Related analysis table (blank indicates that the value under this item does not exist).

**Research facet**	**α**	**Physical education**	**Sports participation**	**Health promotion**
Physical education	0.84			
Sports participation	0.86	0.31^**^		
Health promotion	0.92	0.26^**^	0.41^**^	

** *p-value < 0.01*.

### LISREL Mode Evaluation Indicator

This study summarizes the data results as shown below. The following is a description of the basic fit, internal fit, and overall fit for the model.

According to the results of complete model analysis in Table 3, in terms of basic fit, the three factors of physical education (course design, teaching content, activity design) have reached a significant level in the interpretation of physical education (*t* > 1.96, *p* < 0.05); in the three factors of sports participation (physiological factors, psychological factors, social factors) in the interpretation of sports participation, they have reached a significant level (*t* > 1.96, *p* < 0.05); in the health promotion two factors (physiological health, mental health, practical ideas) in the interpretation of health promotion, they have reached a significant level (*t* > 1.96, *p* < 0.05). It can be seen that the overall model of this study has a good basic fit.

**Table 3:** Overall linear structure pattern analysis results table.

**Assessment item**	**Parameter/rating standard**		**Results**
Basic fit	Physical education	Course design	0.644^**^
		Teaching content	0.672^**^
		Activity design	0.653^**^
	Sports participation	Physiological factors	0.694^**^
		Psychological factors	0.668^**^
		Social factors	0.721^**^
	Health promotion	Physiological health	0.703^**^
		Mental health	0.687^**^

** *p-value < 0.01*.

According to Table 4, it can be seen that there is a positive correlation between physical education and sports participation (0.317, *p* < 0.01), and a positive correlation between sports participation and health promotion (0.358, *p* < 0.01). Also, physical education and health promotion (0.264, *p* < 0.01) also have a positive correlation, representing that hypotheses 1, 2, and 3 are supported.

**Table 4:** The correlation between physical education and sports participation.

**Assessment item**	**Parameter/rating standard**	**Results**
Intrinsic fit	Physical education → sports participation	0.317^**^
	Sports participation → health promotion	0.358^**^
	Physical education → health promotion	0.264^**^

** *p-value < 0.01*.

According to Table 5, in terms of overall mode fit, the overall mode fit standard χ^2^/Df is 1.344, less than the standard value of 3 or less, and the RMR value is 0.007, indicating that the χ^2^/DF and RMR result standards are appropriate, and the chi-square value is very sensitive to the sample size. If it is directly determined by this, it is not appropriate. However, the GFI of the overall mode fit standard is 0.964, and AGFI is 0.917, reaching the standard of more than 0.9. The closer the GFI and AGFI values are to 1, the better the mode fit is. Therefore, this model has better matching indicators.

**Table 5:** Overall mode fit.

Overall fit	χ^2^/Df	1.344
	GFI	0.964
	AGFI	0.917
	RMR	0.007

## Discussion

After the relationship between physical education and health promotion for art students is studied in this paper, it is concluded that: (1) The three aspects of course design, teaching content, and activity design have a great influence on physical education, reaching a significant level (*t* > 1.96, *p* < 0.05). It is almost consistent with the research results of Huang et al. (2018). The results show that the design of the whole link of the course will affect the quality of physical education. (2) The three factors in sports participation of physiological factors, psychological factors, and social factors have a huge influence on physical education, reaching a significant level (*t* > 1.96, *p* < 0.05). It is almost consistent with the results of research conducted by Chang and Gu (2018). It also shows that the physical and psychological factors of the participants, as well as environmental factors, will affect the quality of physical education in the process of sports activities. (3) The three aspects of physiological health, mental health, and practical ideas have a huge influence on health promotion, reaching a significant level (*t* > 1.96, *p* < 0.05). (4) There is a positive correlation between physical education and sports participation (0.317, *p* < 0.01), a positive correlation between sports participation and health promotion (0.358, *p* < 0.01), and a positive correlation between physical education and health promotion (0.264, *p* < 0.01). Also, the hypotheses 1, 2, and 3 proposed in the paper are supported. The results of this research show that the students of the art department participate in multiple exercises to promote their physical health. After doing exercises, they can enhance heart and lung function, strengthen blood circulation, lower blood cholesterol, and enhance the flexibility of hands and feet, as well as avoid disability. The positive effects of fitness on the body are reflected on the diseases, including osteoporosis, coronary heart disease, and cancer. Due to the heavy workload of students, they cannot participate in sports at all times, and it is more suitable for them to participate in sports in spare time. The participated sports are mainly recreational or slow-moving sports, that is, low-intensity recreational sports such as walking, jogging, and swimming. Physical education teachers should pay little attention to speeding up the teaching rhythm and reduce the quality of teaching because of insufficient teaching time; the arrangement of teaching content should emphasize on quality instead of quantity, and focus on the students' awareness rather than how much they have learned.

## Conclusions and Suggestions

The main results are that physical education and sports participation are positively correlated, sports participation and health promotion are positively correlated, and physical education and health promotion are positively correlated. According to the results and findings, the following suggestions are put forward for its practicability:

1. Sports participation can improve the body's immune function, reduce the symptoms of chronic diseases, and have positive effects on health promotion. Art students can actively participate in sports clubs to do sports, such as table tennis, wood balls, croquet, earth dance, and ground golf. Through the leadership of the group, it is easier to benefit from sports participation and stimulate the willingness to participate in sports continuously.
2. It is recommended that art students should develop a healthy lifestyle at an early age, and pay great attention to sports, nutrition, and interpersonal relationships. When they are old, a healthy lifestyle can help maintain their past roles as much as possible, not because of physical, psychological, or social interpersonal relationships. This is because unbalance will lead to various health problems.
3. Physical education teachers should actively participate in the research and training related to physical education, thereby enhancing the professional knowledge of physical education and teaching. Through their own physical education, the teachers may acquire the correct health promotion knowledge of art students, set a model for students, and then implement the teaching of physical education on campus.
4. As a member of the community organization, the school should invest more in community advocacy, marketing, and professional capacity construction, and make use of community organizations to integrate community resources and promote the participation of community residents in sports, which will help students and community health.

This research result enhances the attention of art students to sports. However, the influencing factors are only discussed from a macro perspective (physiological, psychological, and social aspect), which is relatively broad. In the following research, a more systematic analysis from these three perspectives will be carried out to find out the effective ways to promote the sports participation of art students.

## Data Availability Statement

The raw data supporting the conclusions of this article will be made available by the authors, without undue reservation.

## Ethics Statement

The studies involving human participants were reviewed and approved by Wenzhou University Ethics Committee. The patients/participants provided their written informed consent to participate in this study. Written informed consent was obtained from the individual(s) for the publication of any potentially identifiable images or data included in this article.

## Author Contributions

All authors listed have made a substantial, direct and intellectual contribution to the work, and approved it for publication.

## Conflict of Interest

The authors declare that the research was conducted in the absence of any commercial or financial relationships that could be construed as a potential conflict of interest.
